# Electrochemical synthesis of urea on MBenes

**DOI:** 10.1038/s41467-021-24400-5

**Published:** 2021-07-02

**Authors:** Xiaorong Zhu, Xiaocheng Zhou, Yu Jing, Yafei Li

**Affiliations:** 1 Jiangsu Collaborative Innovation Centre of Biomedical Functional Materials, School of Chemistry and Materials Science, Nanjing Normal University, Nanjing, China; 2 College of Chemical Engineering, Nanjing Forestry University, Nanjing, China

**Keywords:** Electrocatalysis, Density functional theory, Electrocatalysis

## Abstract

Urea is an important raw material in the chemical industry and is widely used as a nitrogen source in chemical fertilizers. The current industrial urea synthesis not only requires harsh reaction conditions, but also consumes most of the NH_3_ obtained through artificial synthesis. The conversion of N_2_ and CO_2_ into urea through electrochemical reactions under ambient conditions represents a novel green urea synthesis method. However, the large-scale promotion of this method is limited by the lack of suitable electrocatalysts. Here, by means of density functional theory computations, we systematically study the catalytic activity of three experimentally available two-dimensional metal borides (MBenes), Mo_2_B_2_, Ti_2_B_2_, and Cr_2_B_2_ toward simultaneous electrocatalytic coupling of N_2_ and CO_2_ to produce urea under ambient conditions. According to our results, these three MBenes not only have superior intrinsic basal activity for urea formation, with limiting potentials ranging from −0.49 to −0.65 eV, but also can significantly suppress the competitive reaction of N_2_ reduction to NH_3_. In particular, 2D Mo_2_B_2_ and Cr_2_B_2_ possess superior capacity to suppress surface oxidation and self-corrosion under electrochemical reaction conditions, rendering them relatively promising electrocatalysts for urea production. Our work paves the way for the electrochemical synthesis of urea.

## Abstract (web summary)

The conversion of N_2_ and CO_2_ into urea through electrochemical reactions under ambient conditions represents a novel green urea synthesis method. Here, the authors demonstrate that two-dimensional transition metal borides can serve as effective catalysts for electrochemical urea synthesis.

## Introduction

Urea, also known as carbamide (CO(NH_2_)_2_), was the first organic compound produced from inorganic raw materials. Because its nitrogen content is high (46%) and is readily converted to ammonia (NH_3_) in the soil, urea is now the most commonly used nitrogen fertilizer in the world, and more urea is manufactured by mass than any other organic chemical^1^. While over 90% of produced urea is used as fertilizer, it also has important applications in other fields. For example, urea is a raw material for the manufacture of urea-formaldehyde and urea-melamine-formaldehyde resins^2^. Urea-containing creams are used as topical dermatological products to promote skin hydration^3^, and large amounts of urea are used for the synthesis of barbiturates^4^. Since NH_3_ produced by the hydrolysis of urea can react with nitrogen oxides (NO_x_) to produce nitrogen, an increasingly important application of urea is to reduce NO_x_ impurities in exhaust gases from diesel and lean-burn natural gas engines^5^. Therefore, maintaining a sustainable and efficient urea industry is of great importance for the development of human society.

At present, the production of urea in the industry is accomplished mainly through the reaction of NH_3_ and CO_2_ under high temperature and high pressure. However, this method is not only relatively energy-consuming but also relies on some complex types of equipment and multicycle processes to improve the conversion efficiency^6,7^. Remarkably, urea production consumes ~80% of the global NH_3_, which is mainly derived from the artificial nitrogen reduction reaction (NRR)^8^. Nevertheless, N_2_ is a stable molecule, and substantial input energy is required for dissociation of the strong N≡N triple bond, making N_2_ reduction thermodynamically and kinetically very difficult^9^. The industrial NRR is still dominated by the traditional Haber–Bosch process, which converts N_2_ and H_2_ into NH_3_ with the assistance of iron-based catalysts under harsh conditions^10,11^. However, the very large energy consumption and the large amount of the greenhouse gas CO_2_ emitted by the Haber–Bosch process have aggravated the energy and environmental problems. Therefore, people have been striving for green ammonia synthetic techniques that can be carried out under mild conditions^12–14^.

Compared to the Haber-Bosch process, the production of NH_3_ through the electrochemical NRR represents a more efficient and green strategy as it can utilize electricity generated from renewable energy sources and protons directly from water^15–20^. However, separating and purifying NH_3_ from an aqueous electrolyte is very difficult, which is detrimental to its further application. Furthermore, most current studies mainly focus on N_2_ electrochemical reduction to NH_3_, while further processing of the product is rarely considered. Recently, Jouny et al.^21^ realized C−N coupling and the production of acetamides with a high rate and selectivity by using NH_3_ as a nitrogen source. More interestingly, Comer et al.^22^ demonstrated that during photocatalytic fixation on the surface of TiO_2_, N_2_ can have a strong interaction with carbon substitution sites that present as surface-bound radicals, implying the feasibility of direct formation of C−N bonds from the coupling of N_2_ and carbon-based reagents. Inspired by these pioneering works, Chen et al.^23^ recently successfully coupled N_2_ and CO_2_ in H_2_O to produce urea using an electrocatalyst consisting of Pd-Cu alloy nanoparticles on TiO_2_ nanosheets, which opens a new avenue for urea production under mild conditions. However, in addition to the high price, precious metal-based alloy catalysts usually suffer from ambiguous active sites and easy corrosion. Therefore, for the development of emerging electrochemical urea synthesis, inexpensive and efficient electrocatalysts that can fix N_2_ and CO_2_ together are highly desirable.

In the past decade, the application of two-dimensional (2D) materials in the field of electrocatalysis have received much attention due to their large specific surface area and more exposed active sites^24,25^. For example, Li et al.^26^ theoretically demonstrated that 2D transition metal carbides, namely MXenes, are capable of catalyzing the conversion of CO_2_ into hydrocarbons. However, the surface metal atoms of MXenes are easily passivated by some functional species (e.g. OH, F, O), thereby degrading the catalytic activity^27–29^. Recently, several 2D transition metal borides (MBenes), which are boron analogs of MXenes, have also been realized experimentally^30–33^. In contrast to MXenes, MBenes can be stabilized without the presence of surface passivation groups. Therefore, MBenes provide an ideal platform for the exploration of the catalytic behavior of boron-containing surfaces^34–36^. Some recent theoretical studies have demonstrated that several types of MBenes could present good activity and selectivity for the NRR^37,38^. Motivated by the regulated layered configuration and excellent electrical conductivity of MBenes, we wondered whether the direct coupling of N_2_ and CO_2_ to produce urea could be realized on some specific MBenes.

In this work, by means of density functional theory (DFT) computations, we show that the electrochemical synthesis of urea on the basal planes of three experimentally realized MBenes, including 2D Mo_2_B_2_, Ti_2_B_2_, and Cr_2_B_2_ is thermodynamically and kinetically favorable. Remarkably, the competitive NRR can be significantly overwhelmed on these three MBenes, suggesting good selectivity. Especially, the surface oxidation/degradation problem can be avoided on the surfaces of Mo_2_B_2_ and Cr_2_B_2_, endowing these two MBenes with intrinsic activity, selectivity, and a large reaction region toward electrochemical urea synthesis.

## Results

### Structural properties and stability of MBenes

**Fig. 1:**
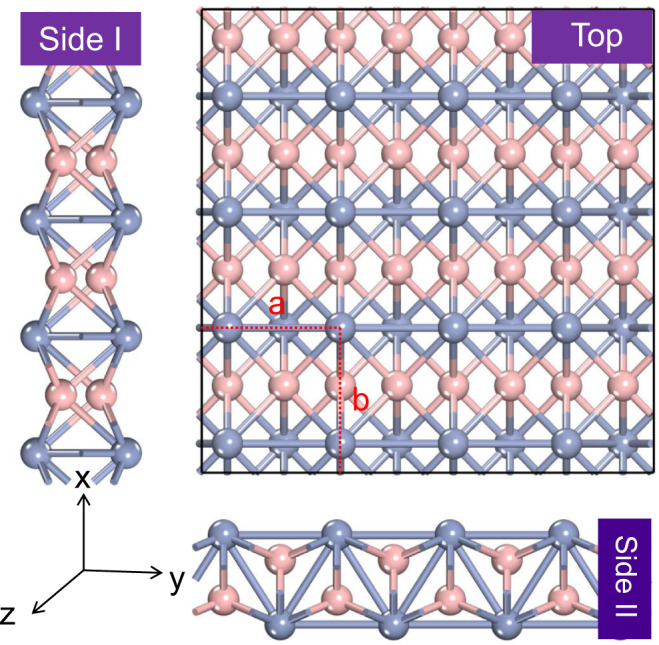
Geometrics of MBenes. Top and side views of the schematic structure of a M_2_B_2_-type MBene. The red dashed lines denote a unit cell. The pink and blue balls represent boron and transition metal atoms, respectively.

Figure 1 shows the schematic structure of a 2D M_2_B_2_-type MBene. Unlike many ordinary 2D materials (e.g., graphene, MoS_2_, MXenes) that possess a hexagonal lattice, our three studied 2D MBenes all present a rectangular lattice with the space group *Pmma* (No. 51), resulting in an in-plane structural anisotropy. The unit cell of a 2D M_2_B_2_-type MBene consists of two metal atoms and two B atoms, and each metal atom or B atom is connected to six neighboring atoms, forming a buckled bilayer structure with metal atoms on the uppermost surface. Compared to MXenes, in which the surface metal atoms bind to three carbon or nitrogen atoms, the higher coordination number of surface metal atoms in M_2_B_2_-type MBenes endow them with distinct stability and properties. The optimized lattice parameters and representative bond lengths of Mo_2_B_2_, Ti_2_B_2_, and Cr_2_B_2_ MBenes are summarized in Supplementary Table 1. Moreover, the electronic band structure computations demonstrated that these three MBenes are all metallic (Supplementary Fig. 1), which is beneficial for their electrocatalytic activity. The above results achieved good agreement with previous studies^39,40^.

Good stability is a prerequisite for the wide utilization of a catalyst. We thus first assessed the stability of 2D Mo_2_B_2_, Ti_2_B_2_, and Cr_2_B_2_ MBenes before revealing their catalytic activity toward urea formation. As shown in Supplementary Fig. 2, no imaginary modes are found in the phonon spectra of the three MBenes, which is indicative of good kinetic stability. The elastic constants of the three monolayers presented in Supplementary Table 2 all meet the criteria for a rectangular 2D structure (C_11_C_22_ − C_12_^2^ > 0, C_66_ > 0)^39^, indicating that they are mechanically stable. Moreover, we also performed first-principles molecular dynamics (FPMD) simulations to examine the thermal stability. According to the diagrams presented in Supplementary Fig. 3, no obvious structural deformation can be observed in the structures of the three MBenes after 10 ps FPMD simulations at 300 K, suggesting good thermal stability.

### Mechanism of electrochemical urea synthesis

**Fig. 2:**
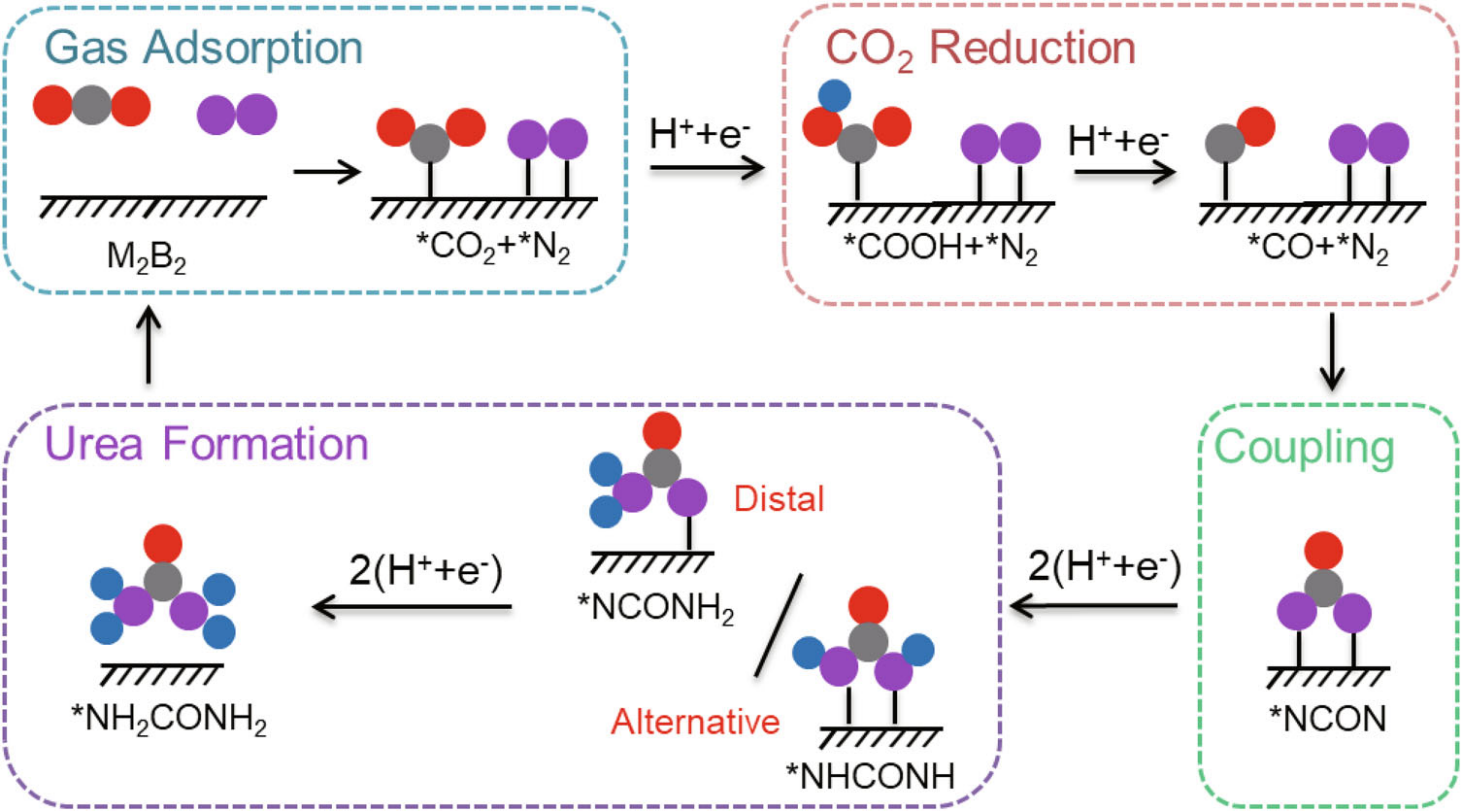
Reaction mechanism. Schematic diagram of the mechanism of urea production through the electrochemical coupling of N_2_ and CO_2_. The gray, red, pink, and blue balls represent C, O, N, and H atoms, respectively.

**Fig. 3:**
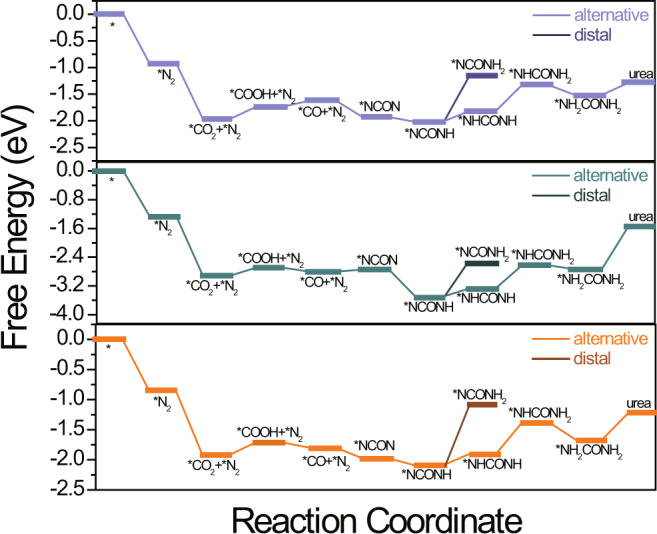
Catalytic activity of MBenes. Free energy profiles of electrochemical urea production on Mo_2_B_2_, Ti_2_B_2_, and Cr_2_B_2_.

To date, the reaction mechanism for electrochemical N_2_ and CO_2_ coupling to produce urea has only been proposed by Chen et al.^23^. As shown in Fig. 2, the entire reaction can be divided into four stages, namely, the adsorption of N_2_ and CO_2_, the reduction of *CO_2_ to *CO, the coupling of *N_2_ and *CO into *NCON, and the hydrogenation of *NCON to urea. Specifically, effective adsorption of N_2_ and CO_2_ on the surface of the catalyst is the primary condition for electrochemical urea production. Moreover, the adsorbed N_2_ should not be reduced to NH_3_, or at least the limiting potential for urea formation (${U}_{L}^{{\rm{urea}}}$) should be lower than that of NH_3_ formation (${U}_{L}^{{{\rm{NH}}}_{3}}$) to guarantee a high selectivity. Once N_2_ is adsorbed, the coadsorbed CO_2_ should be effectively and selectively reduced to *CO. Then, instead of being released or further reduced, the generated *CO should move to the top of adsorbed *N_2_ to form the tower-like key intermediate *NCON. Finally, the formed *NCON species could be further reduced to urea via four proton-coupled electron transfer (PCET) steps following either the distal or the alternative pathway. This mechanism has been well applied to explain the catalytic activity of the Pd-Cu catalyst. For the investigation of urea formation over the three M_2_B_2_-type MBenes, we followed the above-discussed reaction mechanism. The free-energies of reaction intermediates (Supplementary Table 3) and the reaction free-energies (Δ*G*) of elementary steps for urea production on 2D Mo_2_B_2_, Ti_2_B_2_, and Cr_2_B_2_ were then computed. The optimized atomic configurations of various intermediates along the reaction pathway are displayed in Supplementary Fig. 4, and the corresponding free energy profiles are summarized in Fig. 3.

### Electrochemical reactivity of MBenes toward urea production

We first investigated the adsorption of N_2_ and CO_2_ on the surfaces of the three MBenes. For the adsorption of N_2_, both side-on and end-on configurations were considered. According to our computations, the side-on adsorption of N_2_ on the bridge site of two metal atoms is preferred for all three MBenes (Supplementary Fig. 4). This is because the back-donation of electrons from the *d*-orbitals of metal atoms to the π* orbitals of N_2_ could be facilitated by side-on adsorption. The adsorption energies of N_2_ (${\triangle E}_{{\text{N}}_{2}}$) on 2D Mo_2_B_2_, Ti_2_B_2_, and Cr_2_B_2_ are −0.93, −1.27, and −0.85 eV, respectively. Due to the strong interaction between N_2_ and the three MBenes, the bond length of N_2_ is enlarged from 1.10 Å to 1.21, 1.22, and 1.20 Å on 2D Mo_2_B_2_, Ti_2_B_2_, and Cr_2_B_2_, respectively. Once N_2_ is adsorbed, the accommodation of a CO_2_ molecule on its neighboring bridge site is also feasible, with adsorption energies (${\triangle E}_{{\text{CO}}_{2}}$) of −1.04, −1.65, and −1.07 eV for 2D Mo_2_B_2_, Ti_2_B_2_, and Cr_2_B_2_, respectively. Remarkably, the adsorbed CO_2_ is pronouncedly bent due to the formation of chemical bonds between C and metal atoms. Overall, the above results vividly reveal that both N_2_ and CO_2_ can be strongly adsorbed and effectively activated on the surface of our three chosen MBenes.

After disclosing the adsorption behavior of N_2_ and CO_2_, we next assessed the feasibility of *CO_2_ reduction to *CO on the surface of the three MBenes in the presence of coadsorbed N_2_. Generally, the electroreduction of *CO_2_ to *CO initiates with the hydrogenation of one O atom through a PCET step, resulting in the formation of a *COOH species. According to our computations, this step is endothermic with a Δ*G* of 0.24, 0.22, and 0.21 eV for 2D Mo_2_B_2_, Ti_2_B_2_, and Cr_2_B_2_, respectively. Upon the second PCET, the *COOH species can be transformed to a *CO species by releasing a H_2_O molecule, which is endothermic by 0.10 eV for 2D Mo_2_B_2_ but exothermic by −0.11 and −0.08 eV for 2D Ti_2_B_2_ and Cr_2_B_2_, respectively. Therefore, the key intermediate *CO can be feasibly generated on the surfaces of the three MBenes. The adsorption energies of *CO species on 2D Mo_2_B_2_, Ti_2_B_2_, and Cr_2_B_2_ are −0.69, −1.53, and −0.94 eV, respectively, which are all higher than that of Pd-Cu catalyst (−0.62 eV)^23^. Due to the low CO yield of Pd-Cu catalyst, we can expect that the *CO desorption can be significantly suppressed for three 2D MBenes, which could facilitate the following coupling process.

As revealed by Chen et al.^23^ the most important intermediate for urea formation is the tower-like *NCON species, which can be directly produced from the coupling of *CO and *N_2_. We thus explored the feasibility of forming the *NCON species on the surfaces of the three MBenes using the CI-NEB method. As shown in Supplementary Fig. 5, the coupling of *N_2_ and *CO into *NCON is exothermic by −0.30 and −0.18 eV on 2D Mo_2_B_2_ and Cr_2_B_2_, respectively, indicating that the formation of the *NCON species on these two MBenes is energetically favorable. In contrast, due to the relatively strong binding strength of the *CO species, the formation of *NCON on Ti_2_B_2_ is slightly endothermic by 0.06 eV. Remarkably, the kinetic barriers for the formation of *NCON on 2D Mo_2_B_2_, Ti_2_B_2_, and Cr_2_B_2_ are 0.58, 0.81, and 0.71 eV respectively, which are comparable to or even lower than that of the Pd-Cu catalyst (0.79 eV)^23^, indicating that the coupling of *N_2_ and *CO on these three MBenes is kinetically feasible.

Once the *NCON species is formed, the formation of urea becomes very straightforward, although there exist different sequences of hydrogenation on the two N atoms. Remarkably, the hydrogenation of *NCON to *NCONH via a PCET is exothermic for all three MBenes with a Δ*G* of −0.09, −0.76, and −0.12 eV for Mo_2_B_2_, Ti_2_B_2,_ and Cr_2_B_2_, respectively. Notably, we also considered the hydrogenation of the O atom but found that it is relatively endothermic. When the second H is added to *NCONH, two possible reaction pathways may occur. One is the distal pathway in which the second H is added to the hydrogenated N atom in the last step to form the *NCONH_2_ species, and the other is the alternative path in which the second H is added to the bare N atom to form *NHCONH species. According to our computations, the formation of the distal product *NCONH_2_ is endothermic by as high as 0.88, 0.93, and 1.02 eV for 2D Mo_2_B_2_, Ti_2_B_2,_ and Cr_2_B_2_, respectively. In contrast, the formation of the alternative product *NHCONH is 0.68, 0.70, and 0.83 eV lower in energy than that of the distal product for 2D Mo_2_B_2_, Ti_2_B_2,_ and Cr_2_B_2_, respectively. As a comparison, the distal product is preferred by the Pd-Cu catalyst^23^. Additionally, we noted that at this stage, the chemical bonding between metal atoms and N atoms is still robust, while the N−N length in *NHCONH is enlarged to 2.35, 2.34, and 2.33 Å for 2D Mo_2_B_2_, Ti_2_B_2,_ and Cr_2_B_2_, respectively. Due to the breaking of one M−N bond, the third PCET step to form *NH_2_CONH is considerably endothermic, with a Δ*G* of 0.49, 0.65, and 0.52 eV for 2D Mo_2_B_2_, Ti_2_B_2,_ and Cr_2_B_2_, respectively. Since the fourth PCET step to form *NH_2_CONH_2_ is exothermic for all three M_2_B_2_ monolayers, the formation of the *NH_2_CONH species was identified as the potential-limiting step, and the corresponding ${U}_{\text{L}}^{\text{urea}}$ were computed to be −0.49, −0.65, and −0.52 V for Mo_2_B_2_, Ti_2_B_2_, Cr_2_B_2_, respectively. Remarkably, the ${U}_{\text{L}}^{\text{urea}}$ of three MBenes are comparable to or even lower than that of the Pd-Cu catalyst (−0.64 V), which is indicative of superior electrocatalytic reactivity toward urea formation. Moreover, the adsorption energies of *NH_2_CONH_2_ on 2D Mo_2_B_2_, Ti_2_B_2,_ and Cr_2_B_2_ are −1.28, −1.55, and −1.21 eV, respectively, which are lower than that of the Pd-Cu catalyst (−1.68 eV)^23^, indicating that the formed urea molecule can be easily released, especially when electrochemical reactions are carried out in flow cells.

For computational simplicity, the above results on electrochemical steps were all obtained by assuming that the catalyst is charge neutral, and thus, the Fermi-level of the catalyst would change with the variation in adsorbed reaction species. However, in real electrochemical reactions, electron transfer between the catalyst and the electrode to match the Fermi level of the catalyst with the applied electrode potential. In a recent theoretical study, Kim et al.^40^ revealed that the surface charge can have a substantial effect on the electrochemical activity of graphene-based materials. To this end, we also performed grand-canonical DFT computations to investigate the effect of surface charge on the electrocatalytic reactivity of the three MBenes toward urea formation. Supplementary Fig. 6 presents the free energy of all reaction intermediates of urea formation computed using the constant-potential method (CPM), and those computed from the constant-charge method (CCM) are also listed for comparison. Remarkably, the energy difference between the two methods is within the range of 0.1 eV for all three MBenes, which is similar to the values found in bulk materials but much lower than that of graphene. The less pronounced charge effect on MBenes in comparison to graphene should be attributed to greater thickness.

## Discussion

### Electrocatalytic selectivity of CO_2_ reduction on MBenes

The above results demonstrated that the electrochemical production of urea could be feasible on the surfaces of 2D Mo_2_B_2_, Ti_2_B_2_, and Cr_2_B_2_. However, at present we still cannot claim that these three 2D MBenes are qualified catalysts for urea production as some important issues concerning the selectivity are still pending. First, as we discussed above, the *CO species is the key intermediate for the entire reaction. Although we demonstrated that the formation of *CO on the surfaces of the three studied MBenes is feasible via the intermediate *COOH, the transfer of a first proton/electron pair to CO_2_ could also lead to the formation of *OCHO, which is a key intermediate of formate. Therefore, knowing whether CO_2_ can be selectively reduced to *CO is very important. To address this concern, we first computed the binding energy of the *OCHO species on three MBenes, and found that the binding energy of *OCHO is generally higher than the binding energy of *COOH. However, this does not mean that the formation of *OCHO is preferred. As revealed by previous studies^41,42^, many transition metal catalysts bind the *OCHO species more strongly than the *COOH species, but they all have CO, rather than formate, as the major product. Therefore, predicting the selectivity of *CO formation solely based on thermodynamics computations is actually not reliable.

**Fig. 4:**
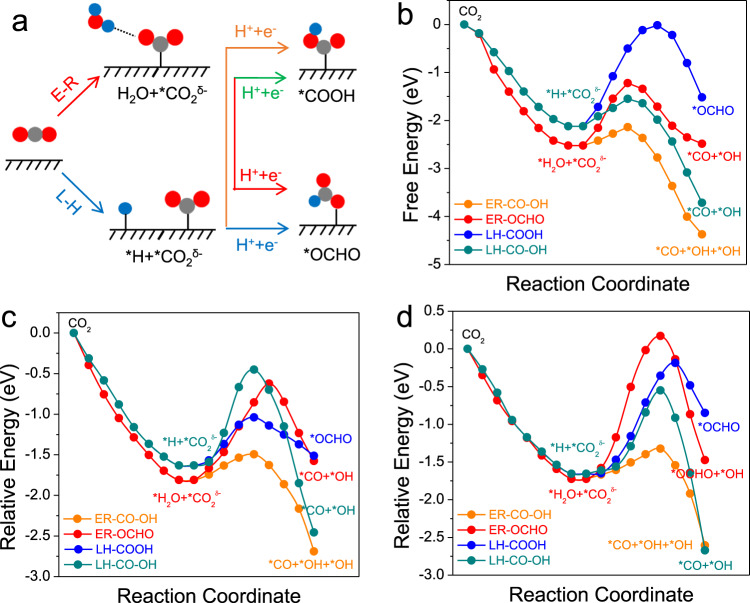
Selectivity of CO_2_ electroreduction. **a** Schematic diagram of the ER and LH mechanisms of CO_2_ electroreduction to *COOH or *OCHO. Kinetic pathways for the electroreduction of CO_2_ on the surfaces of (**b**) Mo_2_B_2_, **c** Ti_2_B_2_, and **d** Cr_2_B_2_.

As revealed by Cheng et al.^43^, the selectivity of CO_2_ reduction is essentially controlled kinetically, and the formation of *COOH and *OCHO can be achieved via either the Eley–Raideal (ER) mechanism by accepting a H atom from water or via the Langmuir-Hinshelwood (LH) mechanism by accepting a surface-bound H atom (Fig. 4a). To obtain some deep insight into the selectivity, we also constructed explicit liquid/solid interfaces for all three MBenes by adding a water layer on their surfaces (Supplementary Fig. 7), and both the ER and LH mechanisms were considered for the formation of *COOH and *OCHO. As shown in Fig. 4b–d, for the formation of the *COOH species, the ER mechanism is preferred with kinetic barriers of 0.38, 0.31, and 0.32 eV for the 2D Mo_2_B_2_, Ti_2_B_2_, and Cr_2_B_2_, respectively. In contrast, the formation of the *OCHO species entails much large kinetic barrier via either the ER mechanism or the LH mechanism. Therefore, on the surfaces of our three chosen 2D MBenes, CO_2_ would be dominantly reduced to *CO rather than formate.

The second concern regarding the *CO species is whether it could be further reduced to *CHO or *COH under the working conditions of urea production. To address this question, we also computed the free energy of *CO reduction to *CHO and *COH for 2D Mo_2_B_2_, Ti_2_B_2_, and Cr_2_B_2_ (Supplementary Table 4). The limiting potential for the formation of *CHO or *COH on the three MBenes is at least −1.22 V, which is much higher than the ${U}_{\text{L}}^{\text{urea}}$. Therefore, the formation of *CHO or *COH would be significantly suppressed under the working potential of urea production.

Moreover, we are also aware of that there would be competition between *CO adsorption and adsorption/dissociation of H_2_O molecule in the realistic aqueous environment. According to our results, the kinetic barrier for the direct dissociation of one H_2_O molecule on 2D Mo_2_B_2_, Ti_2_B_2_, and Cr_2_B_2_ is 0.63, 0.53, and 0.72 eV, respectively (Supplementary Fig. 8), indicating the formation of surface bounded *H and *OH is also feasible for three MBenes. While the *H species can serve as the proton source to react with the reaction intermediates via the LH mechanism, there could exist the adsorption competition between *CO and *OH species on three MBenes. Therefore, we then plotted the curves of equilibrium surface coverages of these two species as a function of electrode potential by performing microkinetic simulations. As shown in Supplementary Fig. 9, the population of *OH and *CO is potential dependent. Specifically, the surfaces of three MBenes would be predominately covered by OH* species under low electrode potential. When the electrode potential is higher than −0.31, −0.64, and −0.22 V, respectively, the coverage of CO* on 2D Mo_2_B_2_, Ti_2_B_2_ and Cr_2_B_2_ begins to increase, while the coverage of OH* begins to decrease. Since the critical potential of *CO/*OH adsorption for each MBene is lower than the respective ${U}_{{\rm{L}}}^{{\rm{urea}}}$, it can be expected that surface active sites of 2D MBenes, especially Mo_2_B_2_ and Cr_2_B_2_, would be mainly covered by *CO rather than *OH under working potentials.

### Electrocatalytic selectivity of N_2_ reduction on MBenes

As reported by previous studies^37,38^, many 2D MBenes have basal plane activity for N_2_ electroreduction to NH_3_. Would the adsorbed N_2_ molecule also be reduced to NH_3_ on our three chosen M_2_B_2_-type MBenes? To address this question, we investigated the thermodynamics of the electrochemical NRR on 2D Mo_2_B_2_, Ti_2_B_2_, Cr_2_B_2_ to get some deep insight. As shown in Supplementary Fig. 10, the electrochemical NRR on all three M_2_B_2_ monolayers is feasible, with ${U}_{\text{L}}^{{\text{NH}}_{3}}$ values of −0.79, −0.71, and −0.65 V, respectively. Encouragingly, for all three MBenes, ${U}_{\text{L}}^{{\text{NH}}_{3}}$ is higher than ${U}_{\text{L}}^{\text{urea}}$, suggesting that the formation of the NH_3_ can be greatly suppressed on these three MBenes.

Since kinetic factors play an important role in determining the selectivity, we further computed the kinetic barriers of elementary steps of N_2_ reduction to urea for three 2D MBenes and compared with those of N_2_ reduction NH_3_. Especially, both ER and LH mechanisms were considered for the electrochemical steps. As presented in Supplementary Figs. 11–13, for all three 2D MBenes, the non-electrochemical step of *NCON formation has the biggest kinetic barrier, which is 0.53, 0.77, and 0.70 eV for Mo_2_B_2_, Ti_2_B_2_, Cr_2_B_2_, respectively. Note that the kinetic barriers of *NCON formation predicted from explicit solvent model are quite close to those predicted from implicit solvent model. As a comparison, the first electrochemical step of N_2_ reduction to NH_3_, namely N_2_ reduction to NNH, already has a relatively big kinetic barrier, which is 0.78, 0.80, and 0.76 for 2D Mo_2_B_2_, Ti_2_B_2_, Cr_2_B_2_, respectively (Supplementary Fig. 14). Therefore, the urea synthesis is also kinetically favorable on these three 2D MBenes.

### Pourbaix diagrams of MBenes

**Fig. 5:**
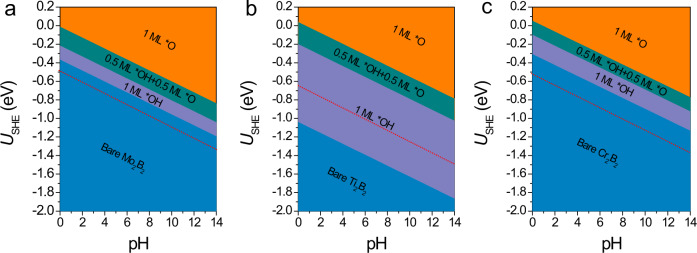
Surface states of MBenes under electrochemical conditions. Surface Pourbaix diagrams of 2D (**a**) Mo_2_B_2_, (**b**) Ti_2_B_2_, and (**c**) Cr_2_B_2_. The thermodynamically stable states of the surface under SHE and pH values are highlighted by orange (for *O), green (for *O + *OH), and purple (for *OH). The red dashed line represents the limiting potential of urea formation.

Finally, another concern that also needs to be addressed is the electrochemical stability of the three MBenes. Although no evidence yet confirm the existence of functional groups on experimentally realized MBenes, we wondered whether the bare surfaces of MBenes could be covered by *O/*OH species in aqueous solution under working conditions. To answer this question, we constructed surface Pourbaix diagrams of the three MBenes to reveal the most stable surface configurations under different equilibrium potentials and pH values (the computational details are given in Supplementary Methods)^44,45^. As shown in Fig. 5, when the electrode potential is 0 V vs SHE, the basal plane of the 2D Mo_2_B_2_ is fully covered by *O species independent of the pH value, whereas the basal planes of the 2D Ti_2_B_2_ and Cr_2_B_2_ are covered by *O and *OH groups in a strong acid environment. When an electrode potential is applied, the hydrogenation of *O and *OH becomes energetically favorable on the surfaces of all three MBenes. In particular, the minimum potentials required to remove the surface *O/*OH species at pH = 0 (${U}_{\text{R}}$) are −0.37, −1.04, and −0.31 V for the 2D Mo_2_B_2_, Ti_2_B_2_, and Cr_2_B_2_ monolayers, respectively. It is worth noting that the ${U}_{\text{R}}$ of 2D Mo_2_B_2_ and Cr_2_B_2_ is less negative than the respective ${U}_{\text{L}}^{\text{urea}}$, indicating that these two MBenes could possess superior electrochemical stability against surface oxidation under working conditions. In sharp contrast, the ${U}_{\text{R}}$ of 2D Ti_2_B_2_ is far more negative than its ${U}_{\text{L}}^{\text{urea}}$ and even ${U}_{\text{L}}^{{\text{NH}}_{3}}$. As the electrode potential of urea production on the 2D Ti_2_B_2_ should not exceed the ${U}_{\text{L}}^{{\text{NH}}_{3}}\,$to guarantee a high selectivity, the surface of the 2D Ti_2_B_2_ would inevitably be occupied by *OH/*O species under working conditions, resulting in decreased reactive sites on the basal plane.

**Fig. 6:**
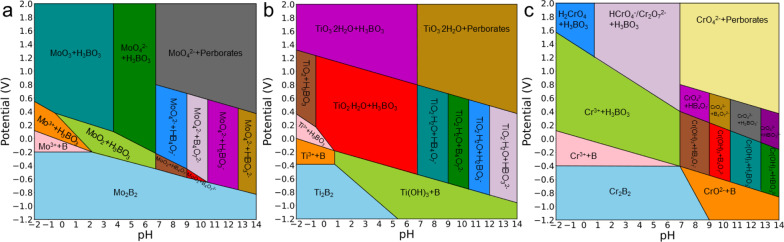
Corrosion resistance of MBenes under electrochemical conditions. Computationally predicted Pourbaix diagrams of (**a**) Mo_2_B_2_, (**b**) Ti_2_B_2_, and (**c**) Cr_2_B_2_ using 10^−6^ M concentration for aqueous species at 25 °C.

Besides the stability of active surfaces, whether the catalyst itself would corrode under electrochemical conditions also needs to be addressed^46,47^. Taking advantage of the fact that the formation energies are transferable between energy reference systems, we further plotted the whole Pourbaix diagram of the three 2D MBenes as a function of pH and potential at standard conditions to identified their stability window in aqueous solutions by directly combining Gibbs free energies from DFT computations with experimental arbitrary aqueous states. As shown in Fig. 6, a wide passivation region can be identified for Mo_2_B_2_ (pH < 7.08) and Cr_2_B_2_ (pH < 7.22) at the potential of −0.49 V and −0.52 V, respectively. Using the electrolyte of Pd–Cu catalyst (pH = 6.8)^23^ as a reference, both 2D Mo_2_B_2_ and Cr_2_B_2_ can maintain structure integrality under working conditions due to high barriers for solid–liquid phase transformations. In sharp contrast to 2D Mo_2_B_2_ and Cr_2_B_2_, 2D Ti_2_B_2_ has a narrow passivation region (pH < 2.26) when subjected to an external potential of −0.65 V. At high pH region, Ti_2_B_2_ would be transformed into Ti(OH)_3_, indicating that Ti_2_B_2_ would be easily corroded under working conditions of urea synthesis, Therefore, 2D Ti_2_B_2_ is not a qualified electrocatalyst for urea formation due to its low electrochemical stability.

### Activity origin of MBenes toward urea production

Due to the good reactivity, selectivity, and stability, 2D Mo_2_B_2_ and Cr_2_B_2_ have been identified as promising electrocatalysts for urea synthesis. However, at present, the activity origin of these two MBenes is not yet clear. It is known that the activity of an electrocatalyst is essentially governed by its electronic structure. Therefore, in order to get some deeper insights into the specialty of MBenes for urea synthesis, we further computed their electronic density of states (DOS) at the Fermi level and compared with those of some other experimentally realized 2D metallic materials, including four bare MXenes (Ti_2_C, Mo_2_C, Ti_3_C_2_, Mo_3_C_2_) and two transition metal chalcogenides (1T-MoS_2_, VS_2_). As shown in Supplementary Fig. 15, the DOS per atom of MXenes are generally much higher than those of 2D Mo_2_B_2_ and Cr_2_B_2_, implying stronger metallicity and more active surfaces of MXenes. However, this does not mean that MXenes have better electrochemical activity than MBenes, because the active surfaces of MXenes are known to be easily passivated in solvents^27–29^. Interestingly, the DOS of Ti_2_B_2_ are quite close to those of Mo_2_C and Mo_3_C_2_, which could explain the instability of Ti_2_B_2_ in aqueous solution. Remarkably, the DOS of Mo_2_B_2_ and Cr_2_B_2_ are significantly higher than those of 1T-MoS_2_ and VS_2_ that are actually inert to urea production according to our test computations. Therefore, the good activity and stability of 2D Mo_2_B_2_ and Cr_2_B_2_ should be attributed to their moderate metallicity.

To summarize, on the basis of comprehensive DFT computations, we have systematically explored the potential of utilizing the three experimentally realized MBenes, Mo_2_B_2_, Ti_2_B_2_, and Cr_2_B_2_, as electrocatalysts for urea synthesis. The activity, selectivity, and stability of the three MBenes under aqueous conditions were carefully studied. Our computations demonstrated that all three MBenes can adsorb N_2_ and CO_2_ on their basal planes, and the adsorbed CO_2_ can be easily reduced to *CO. Afterwards, the key intermediate *NCON can be formed via the coupling of *N_2_ and *CO, which can be further reduced to urea via four PCET steps. The limiting potentials of urea formation for our three studied MBenes are in the range of −0.49 to −0.65 eV, which are comparable to that of the Pd-Cu alloy catalyst. In particular, it is found that 2D Mo_2_B_2_ and Cr_2_B_2_ can prevent the problems of active sites blockage and self-corrosion, while 2D Ti_2_B_2_ not only has its surface active sites occupied by *OH and *O groups, but also could be easily corroded under reaction conditions. Therefore, 2D Mo_2_B_2_ and Cr_2_B_2_ can serve as promising catalysts for urea production, which can be attributed to their moderate metallicity. Our work provides a clear roadmap for the design of electrocatalysts for simultaneously fixing N_2_ and CO_2_ to produce urea, which could promote more experimental and theoretical efforts on developing 2D electrocatalysts for this challenging reaction.

## Methods

### DFT computations

Our DFT computations based on first-principles were performed via the Vienna ab initio simulation package (VASP)^48^. The ion-electron interactions were described with the projector-augmented plane-wave (PAW) method^49^. Exchange-correlation potentials were expressed by Perdew–Burke–Ernzerhof (PBE) functional with the generalized gradient approximation (GGA)^50^. A 460 eV cutoff energy for the plane wave expansion was adopted in all the computations. A Monkhorst-Pack k-points setting of 3 × 3 × 1 and 15 × 15 × 1 was used to sample the 2D Brillouin zone for geometry optimizations and electronic structure computations, respectively. We set the *x* and *y* directions parallel and the *z* direction perpendicular to the layer plane, and adopted a vacuum layer length of 20 Å in the *z* direction. The systems were relaxed until the energy and force reaching the convergence threshold of 10^−5^ eV and 0.01 eV/Å.

The phonon spectra were computed using the density functional perturbation theory (DFPT), as implemented in the Phonon code^51^. The ab initio molecular dynamic (AIMD) simulations were performed using the NVT ensemble. The Nosé-Hoover method simulations last 10 ps with a time step of 1.0 fs^52^. The solvation effects in aqueous solution were considered with the Poisson-Boltzmann implicit solvation model as implemented in VASP (VASP-sol), where the dielectric constant of water was taken as 80^53^. The climbing-image nudged elastic band (CI-NEB) method^54^ as implemented in VASP was used to obtain the kinetic barriers.

The grand-canonical DFT computations, which allow the number of electrons to adjust automatically at a fixed electron chemical potential, were performed using the PBE functional as implemented in JDFTx code^55^ with a cutoff energy of 20 Hartree. The JDFTx code combines electronic DFT with classical DFT and continuum models of liquids for first-principles computations of electrochemical systems. The charge-asymmetric nonlocal determined local electric (CANDLE)^56^ solvation model as implemented in JDFTx was utilized for describing the electrolyte. Other numerical parameters, including k-point sampling, convergence criteria, etc. are similar to the VASP computations.

### Free energy computations

To compute the free energy change (Δ*G*) of each elementary step of electrochemical urea synthesis, we adopted the computational hydrogen electrode (CHE) model developed by Nørskov et al.^57,58^ according to which the Δ*G* of an electrochemical reaction is computed as:

1 $$\varDelta G=\varDelta E+\varDelta {E}_{{\rm{ZPE}}}-T\varDelta S$$

where Δ*E* is the DFT computed reaction energy, Δ*E*_ZPE_ and Δ*S* are the zero-point energy difference and the entropy difference between the adsorbed state and the gas phase, respectively, and *T* is the temperature (298.15 K, in our work). For adsorbed reaction intermediates, their *E*_ZPE_ and *S* are obtained via vibrational frequencies computations with harmonic approximation and neglecting contributions from the slab, while for molecules these are taken from the NIST database. Moreover, in accordance with the CHE model, the effects of electrode potential (*U*) and pH can be treated as an energy shift to free energy change in the electrochemical steps:

2 $$\varDelta {G}_{U}=-eU$$

3 $$\varDelta {G}_{{\rm{pH}}}=-{k}_{B}T\,{\mathrm{ln}}\,10\times {\rm{pH}}$$

where *k*_B_ is Boltzmann constant. In this work, the value of pH was assumed to be zero in free energy computations.

## Supplementary information

Supplementary Information

## Data Availability

The authors declare that the data supporting the findings of this study are available within the paper and its supplementary information files. All of the other data are available from the corresponding author upon reasonable request.

## Code availability

The computational codes used in this work are available from the corresponding author on reasonable request.
