# Supplementary material for: Electrochemical synthesis of urea on MBenes
Source: Nat Commun. 2021 Jul 2;12:4080. doi: 10.1038/s41467-021-24400-5 (PMC8253759; doi:10.1038/s41467-021-24400-5)
Supplement: Supplementary file 1 — Supplementary Information [file 41467_2021_24400_MOESM1_ESM.pdf]

Supplementary Information for

Electrochemical Synthesis of Urea on MBenes

Xiaorong Zhu,[1] Xiaocheng Zhou,[1] Yu Jing,[2] Yafei Li,[1]*

[1]Jiangsu Collaborative Innovation Centre of Biomedical Functional Materials, School of Chemistry and Materials Science, Nanjing Normal University, Nanjing 210023, China

[2] College of Chemical Engineering, Nanjing Forestry University, Nanjing 210037, China

* To whom correspondence should be addressed. Email: liyafei@njnu.edu.cn (YL)

Supplementary Table 1. **Structural properties.** The optimized lattice parameters (*a*, *b*) and the TM−B bond lengths (d_{TM-B}) of 2D Mo_2B_2, Ti_2B_2, and Cr_2B_2.

	a (Å)	*b* (Å)	d_{TM-B} (Å)
Mo_2B_2	3.06	3.05	2.28, 2.26
Ti_2B_2	3.18	2.96	2.30, 2.28
Cr_2B_2	2.95	2.88	2.21, 2.15

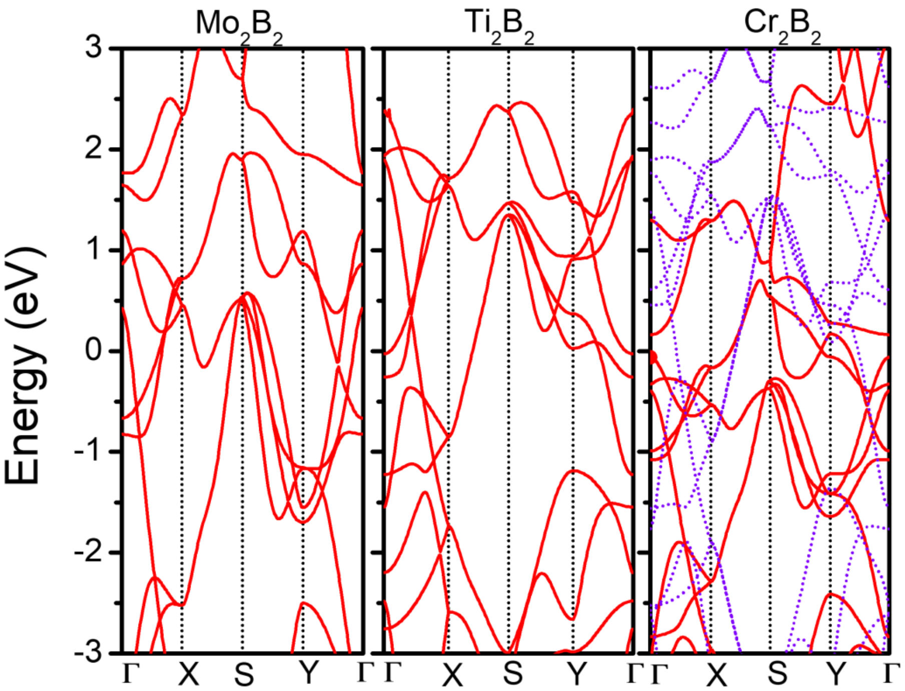

Supplementary Fig 1 Electronic properties. Band structures of 2D Mo_2B_2, Ti_2B_2, and Cr_2B_2. For 2D Cr_2B_2, both spin-up (red) and spin-down (blue) channels are shown. The Fermi level is assigned at zero.

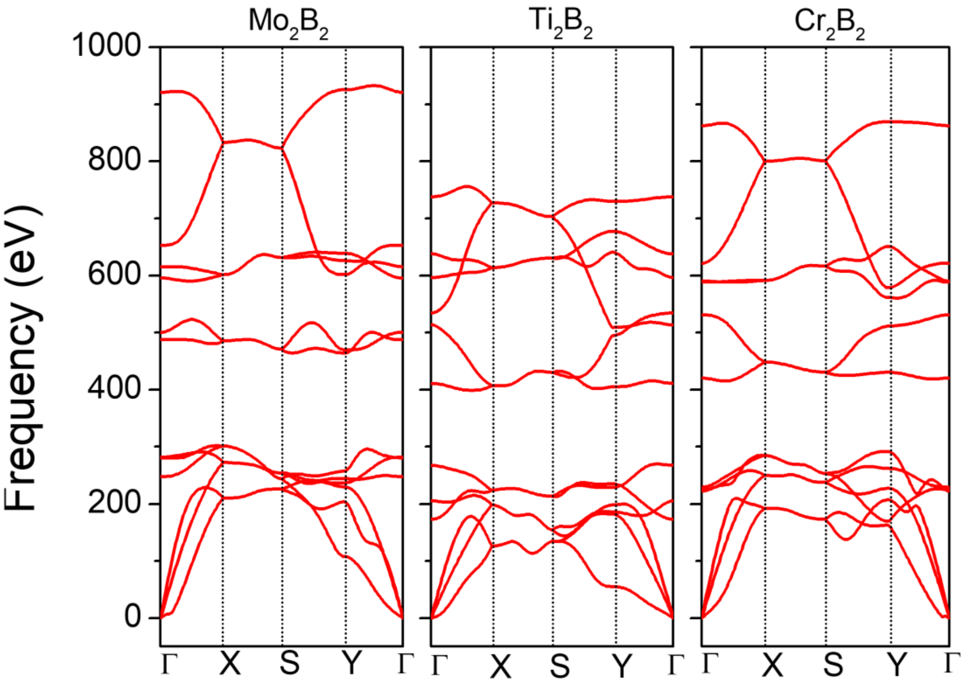

Supplementary Fig. 2 Kinetic stability. Phonon spectra of 2D Mo_2B_2, Ti_2B_2, and Cr_2B_2.

Supplementary Table 2. Mechanical properties. Elastic constants of 2D Mo_2B_2, Ti_2B_2, and Cr_2B_2.

	C_{11} (N/m)	C_{22} (N/m)	C_{12} (N/m)	C_{66} (N/m)
**Mo_2B_2**	206.80	222.18	67.74	80.20
**Ti_2B_2**	97.19	218.16	15.50	93.19
**Cr_2B_2**	208.91	160.88	55.14	96.95

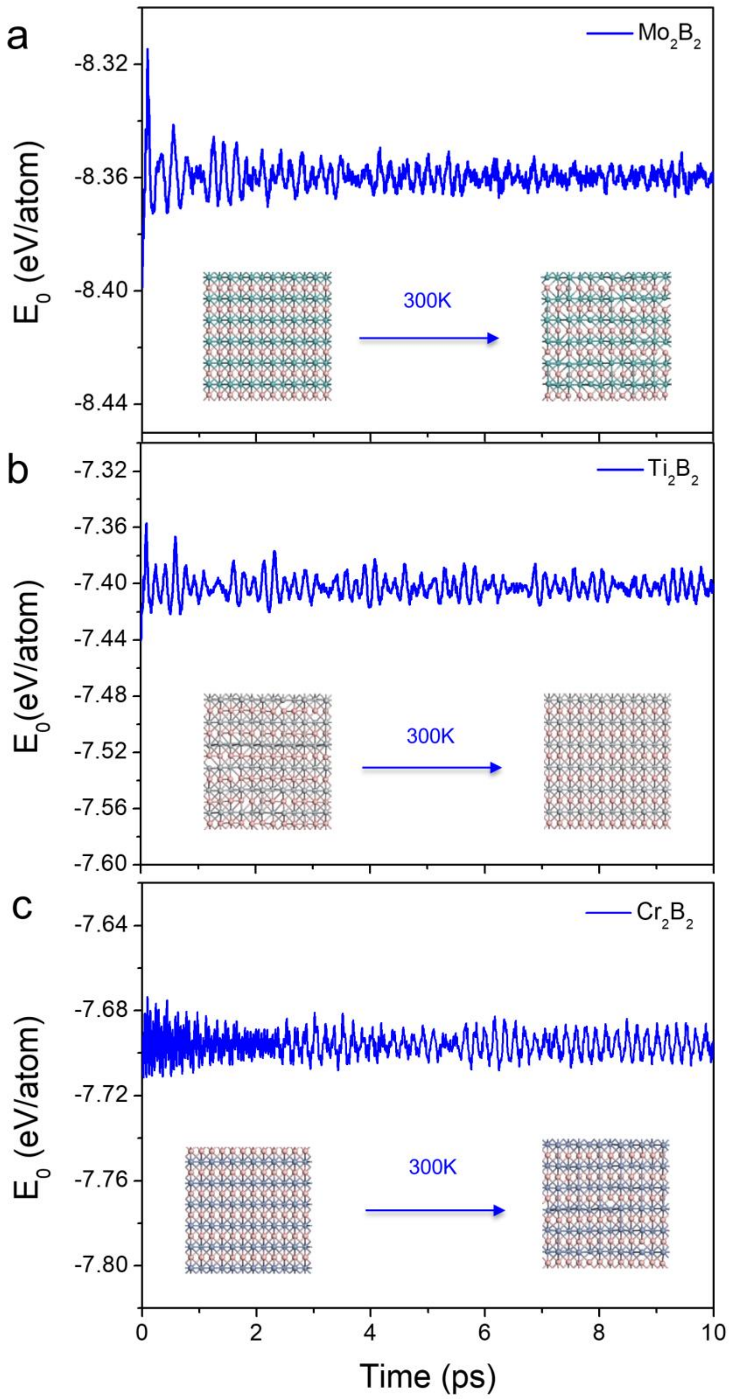

Supplementary Fig. 3 Molecular dynamics simulations. The evolution of the total energy of first-principles molecular dynamics (FPMD) simulations for 2D (a) Mo_2B_2, (b) Ti_2B_2, (c) and Cr_2B_2 at 300 K. The insets are snapshot structures of three MBenes at 0 ps and 10 ps.

Supplementary Table 3. Free energy computations. Computed zero-point energies and entropy of reaction intermediates on the 2D Mo_2B_2, Ti_2B_2, and Cr_2B_2.

**Species**	**ZEP (eV) (Mo_2B_2)**	**TS (eV) (Mo_2B_2)**	**ZEP (eV) (Ti_2B_2)**	**TS (eV) (Ti_2B_2)**	**ZEP (eV) (Cr_2B_2)**	**TS (eV) (Cr_2B_2)**
**$*N_2$**	0.20	0.08	0.19	0.11	0.19	0.11
***NNH**	0.49	0.10	0.46	0.13	0.49	0.10
***NHNH**	0.82	0.10	0.78	0.15	0.82	0.10
**$*NNH_2$**	0.83	0.13	0.81	0.16	0.83	0.13
**$*NHNH_2$**	1.14	0.15	1.12	0.18	1.13	0.15
**$*NH_2NH_2$**	1.50	0.16	1.47	0.18	1.49	0.15
***N**	0.07	0.07	0.07	0.07	0.07	0.06
***NH**	0.40	0.17	0.33	0.11	0.36	0.08
**$*NH_2$**	0.65	0.13	0.63	0.14	0.63	0.19
**$*CO+*N_2$**	0.43	0.17	0.37	0.28	0.37	0.28
***NCON**	0.43	0.17	0.40	0.24	0.43	0.17
***NCONH**	0.76	0.20	0.74	0.23	0.76	0.21
***NHCONH**	1.09	0.22	1.07	0.26	1.08	0.22
**$*NCONH_2$**	1.06	0.21	1.09	0.20	1.08	0.19
**$*NHCONH_2$**	1.37	0.09	1.37	0.09	1.40	0.10
**$*NH_2CONH_2$**	1.68	0.04	1.63	0.06	1.68	0.04

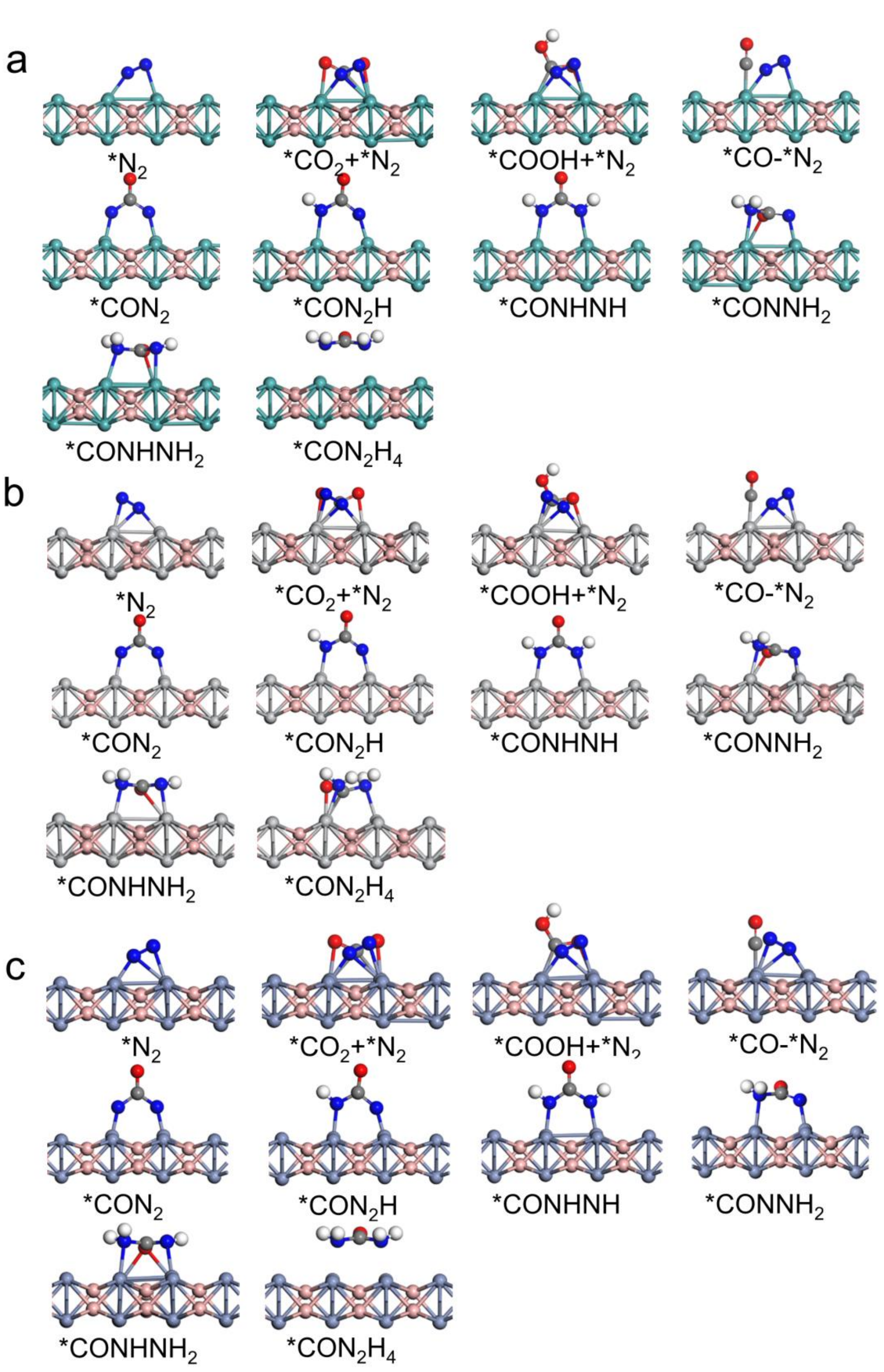

Supplementary Fig. 4 Configurations of reaction intermediates. The optimized geometric structures of various states along the reaction path of urea production on 2D (a) Mo_2B_2, (b) Ti_2B_2, (c) and Cr_2B_2.

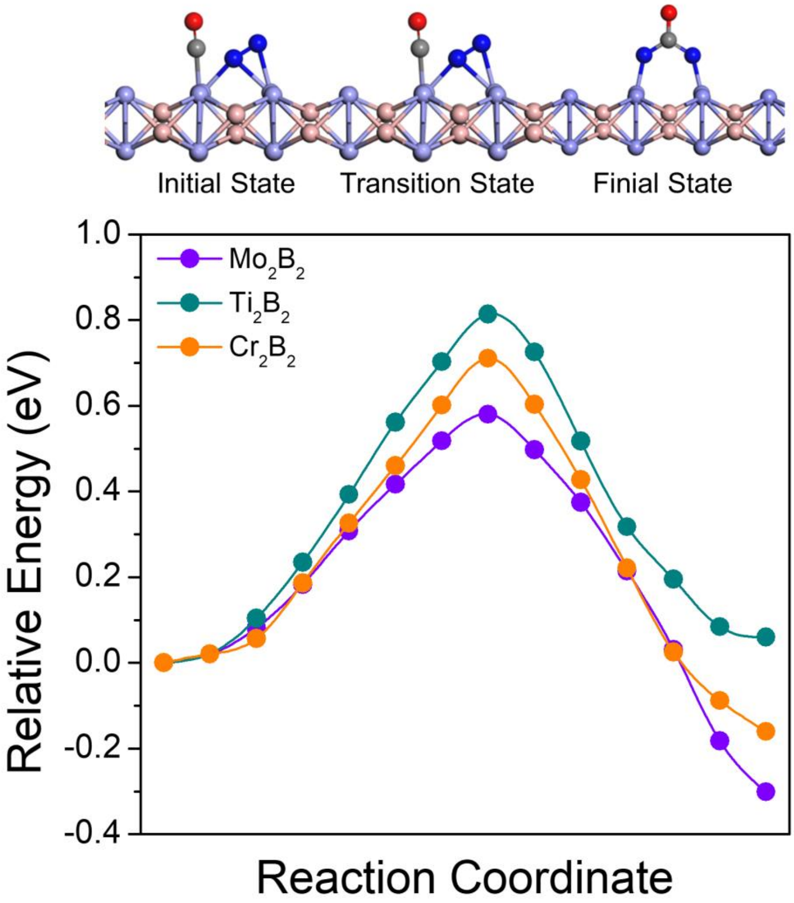

Supplementary Fig. 5 CI-NEB studies of *NCON formation. Representative configurations and corresponding energy barriers along the kinetic pathways of $*N_2$ and *CO coupling into *NCON on 2D Mo_2B_2, Ti_2B_2, and Cr_2B_2.

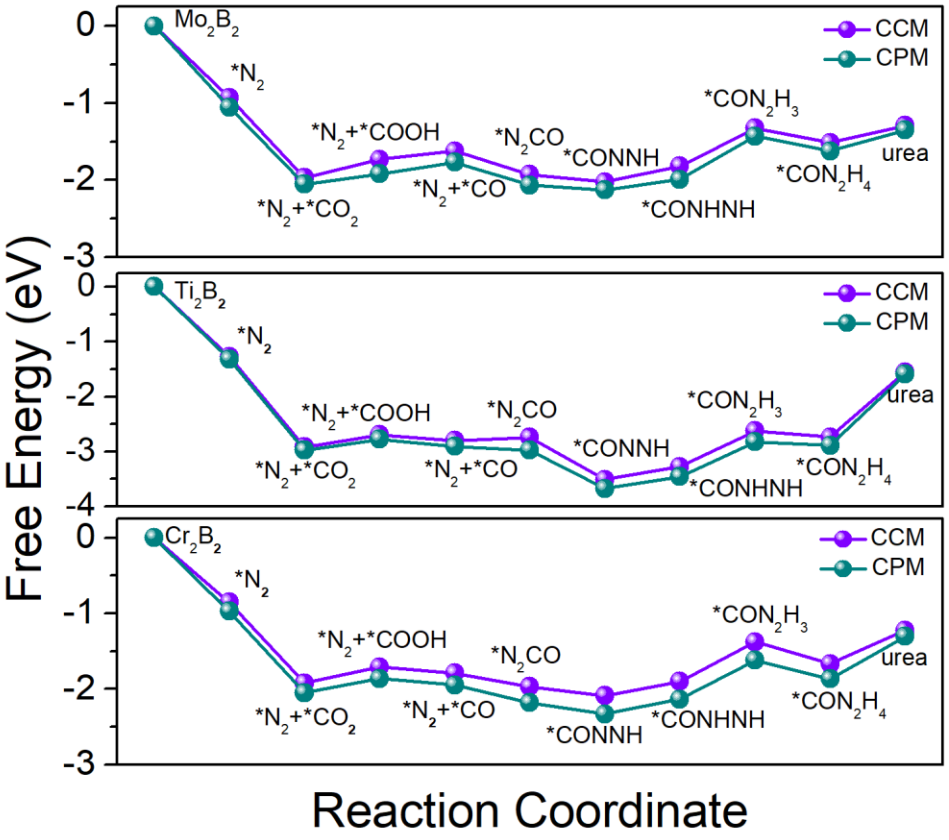

Supplementary Fig. 6 Surface charge effect. Comparison of the free energy evolution of urea formation on 2D Mo_2B_2, Ti_2B_2, and Cr_2B_2 computed from constant charge and constant potential methods.

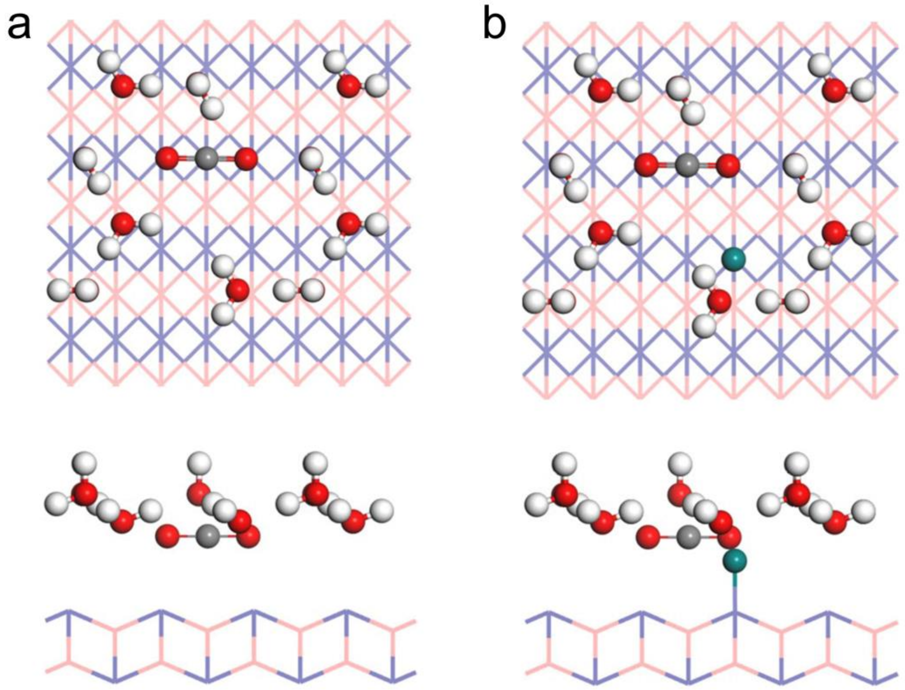

Supplementary Fig. 7 Solvent models. Top and side view of explicit solvent model implemented on 2D MBenes to simulate the (a) ER and (b) LH mechanisms. The red, white, and green balls represent the O, H and surface adopted H atoms respectively.

Supplementary Table 4. Selectivity of CO* reduction. The reaction free energies of *COH (ΔG_{*COH}) and *CHO formation (ΔG_{*CHO}) on 2D Mo_2B_2, Ti_2B_2, Cr_2B_2.

	Mo_2B_2	Ti_2B_2	Cr_2B_2
ΔG_{*COH} (eV)	1.53	1.96	1.35
ΔG_{*CHO} (eV)	1.47	1.78	1.22

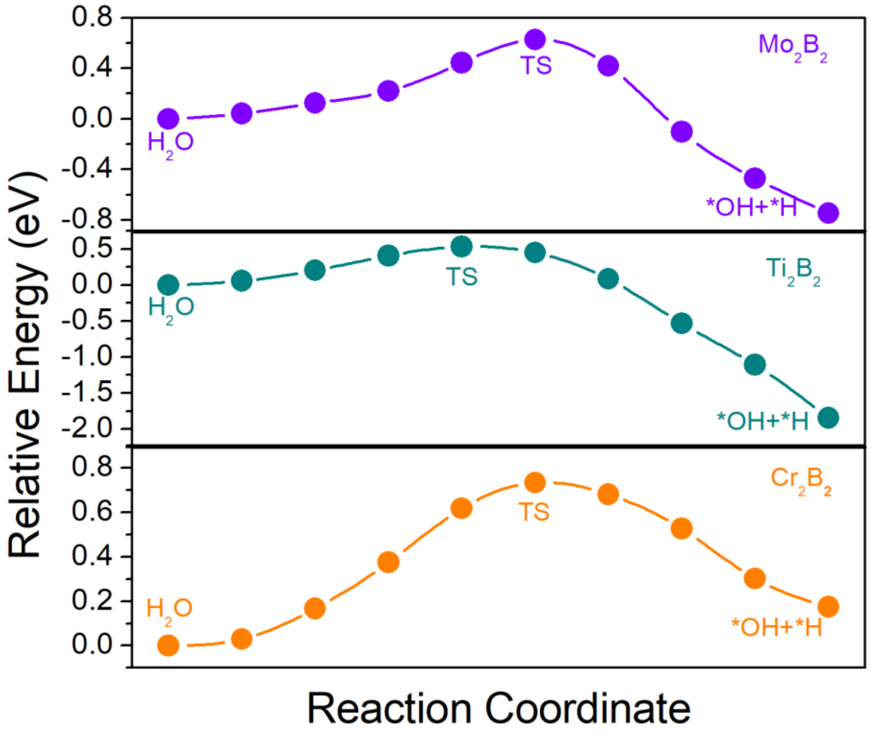

Supplementary Fig. 8 Kinetics of water dissociation. Kinetic pathways of one H_2O molecule dissociation into *H and *OH on 2D Mo_2B_2, Ti_2B_2, and Cr_2B_2.

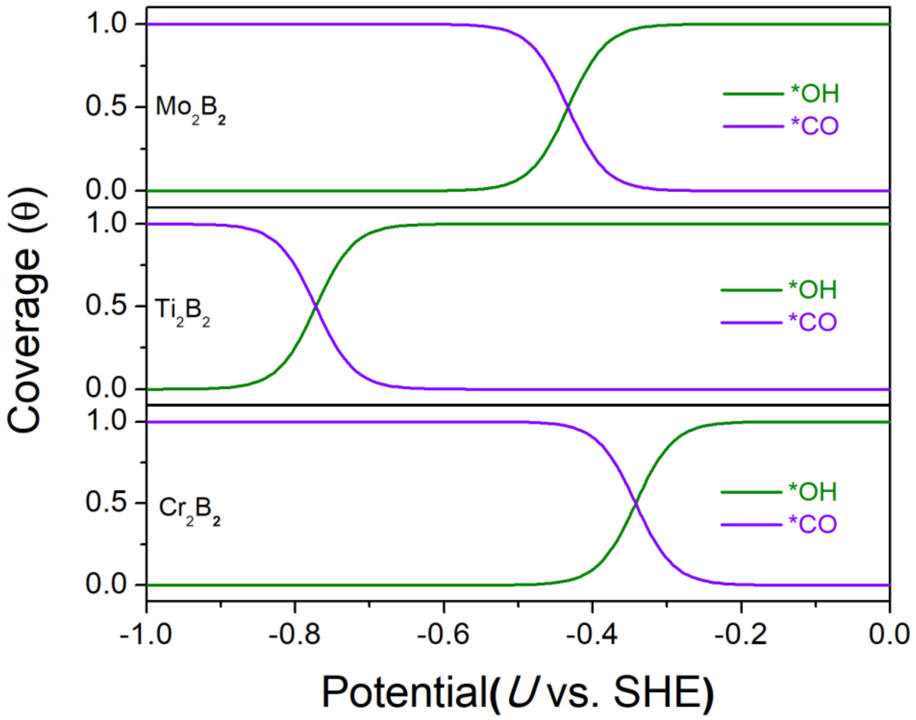

Supplementary Fig. 9 Adsorption competition between *CO and *OH. Coverages of *CO and *OH species on 2D Mo_2B_2, Ti_2B_2 and Cr_2B_2 monolayers as a function of *U*.

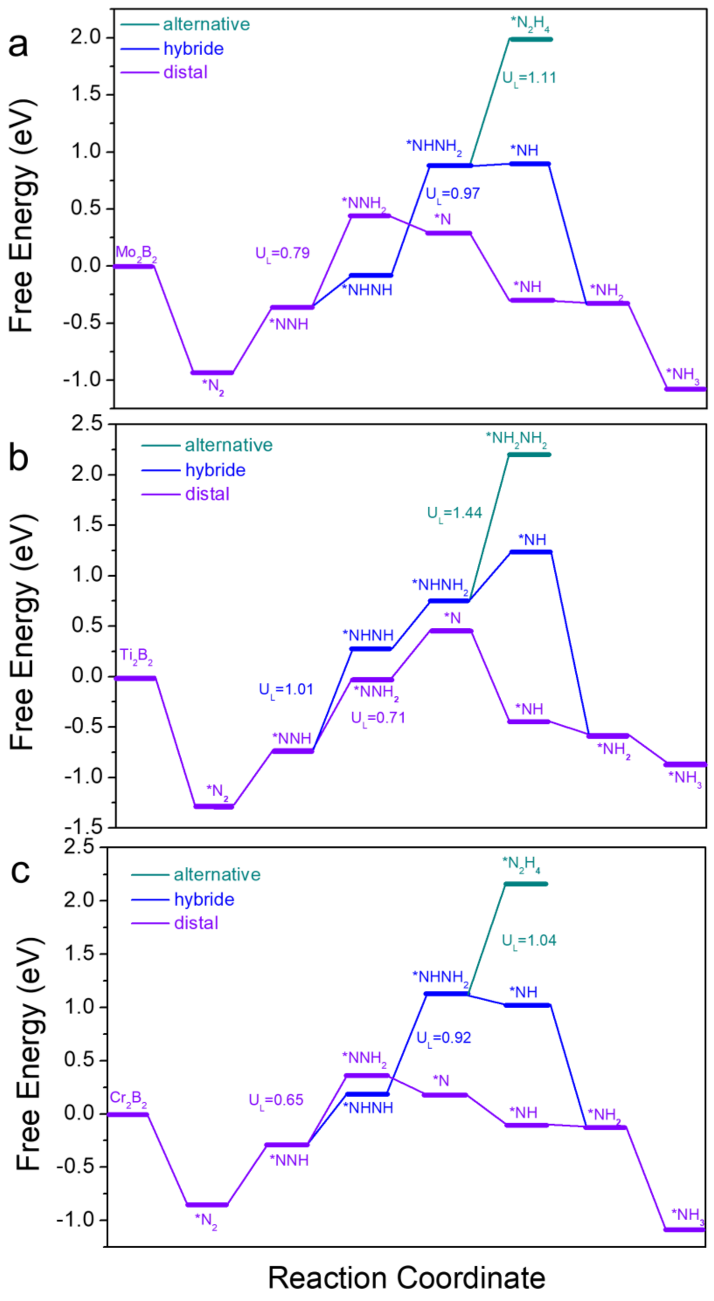

Supplementary Fig. 10 Thermodynamics of N_2 reduction to NH_3. Free energy diagram of NRR on 2D Mo_2B_2, Ti_2B_2, and Cr_2B_2.

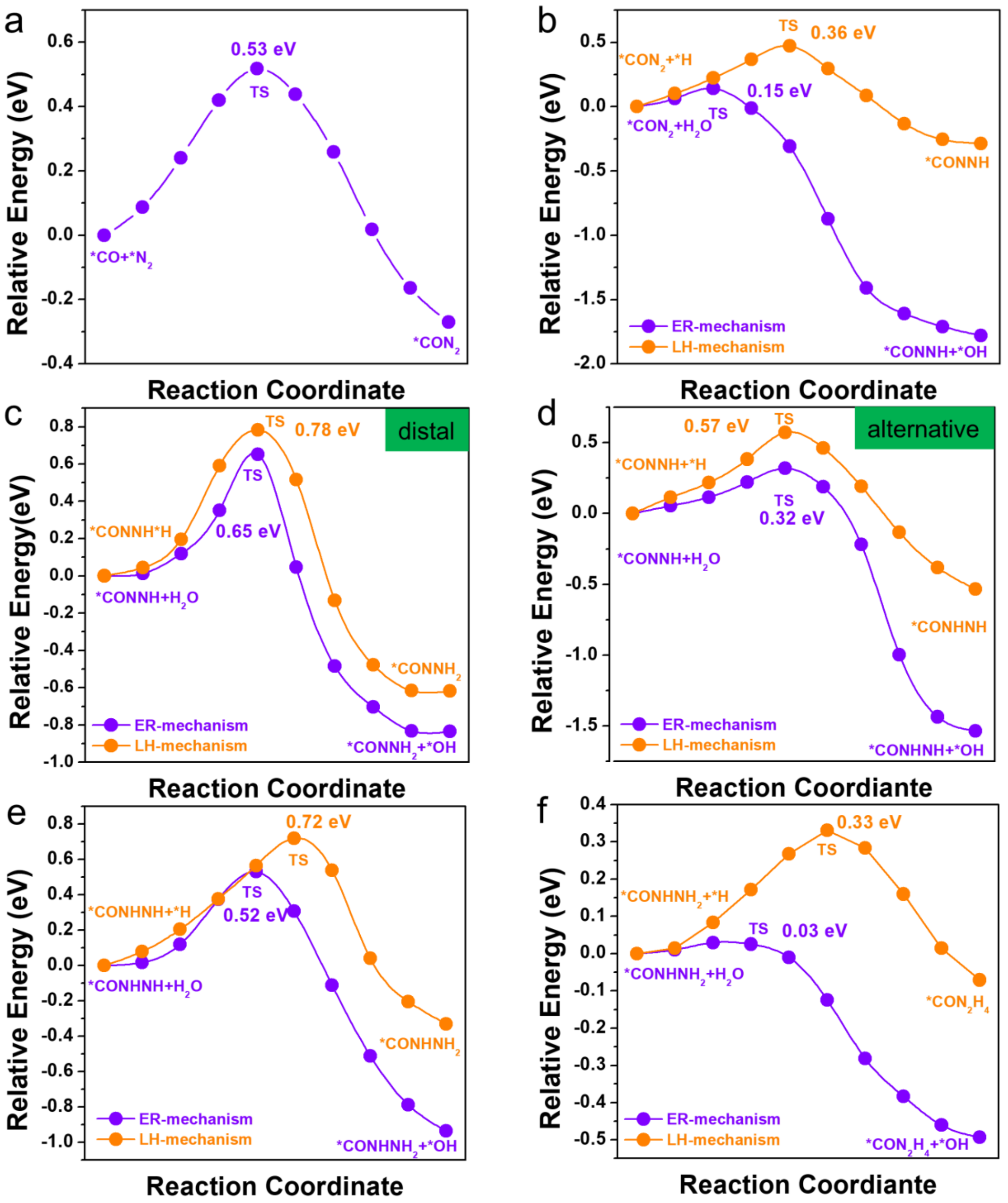

Supplementary Fig. 11 Kinetics of N_2 reduction to urea on Mo_2B_2. Kinetic pathways of elementary steps of N_2 reduction to urea on 2D Mo_2B_2. For electrochemical steps, both LH and ER mechanisms were considered. Especially, for the hydrogenation of *CONNH species, both distal and alternative pathways were considered.

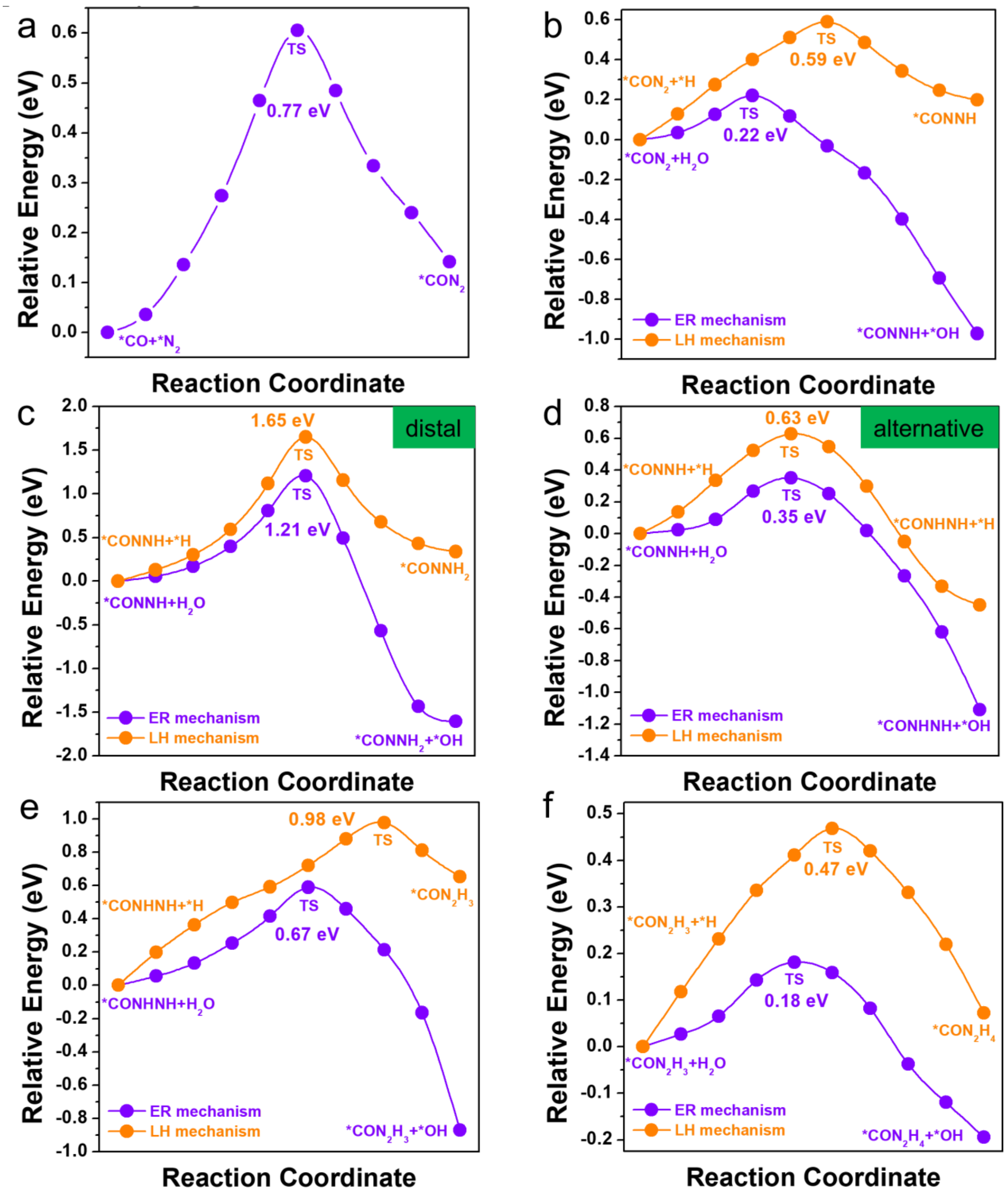

Supplementary Fig. 12 Kinetics of N_2 reduction to urea on Ti_2B_2. Kinetic pathways of elementary steps of N_2 reduction to urea on 2D Ti_2B_2

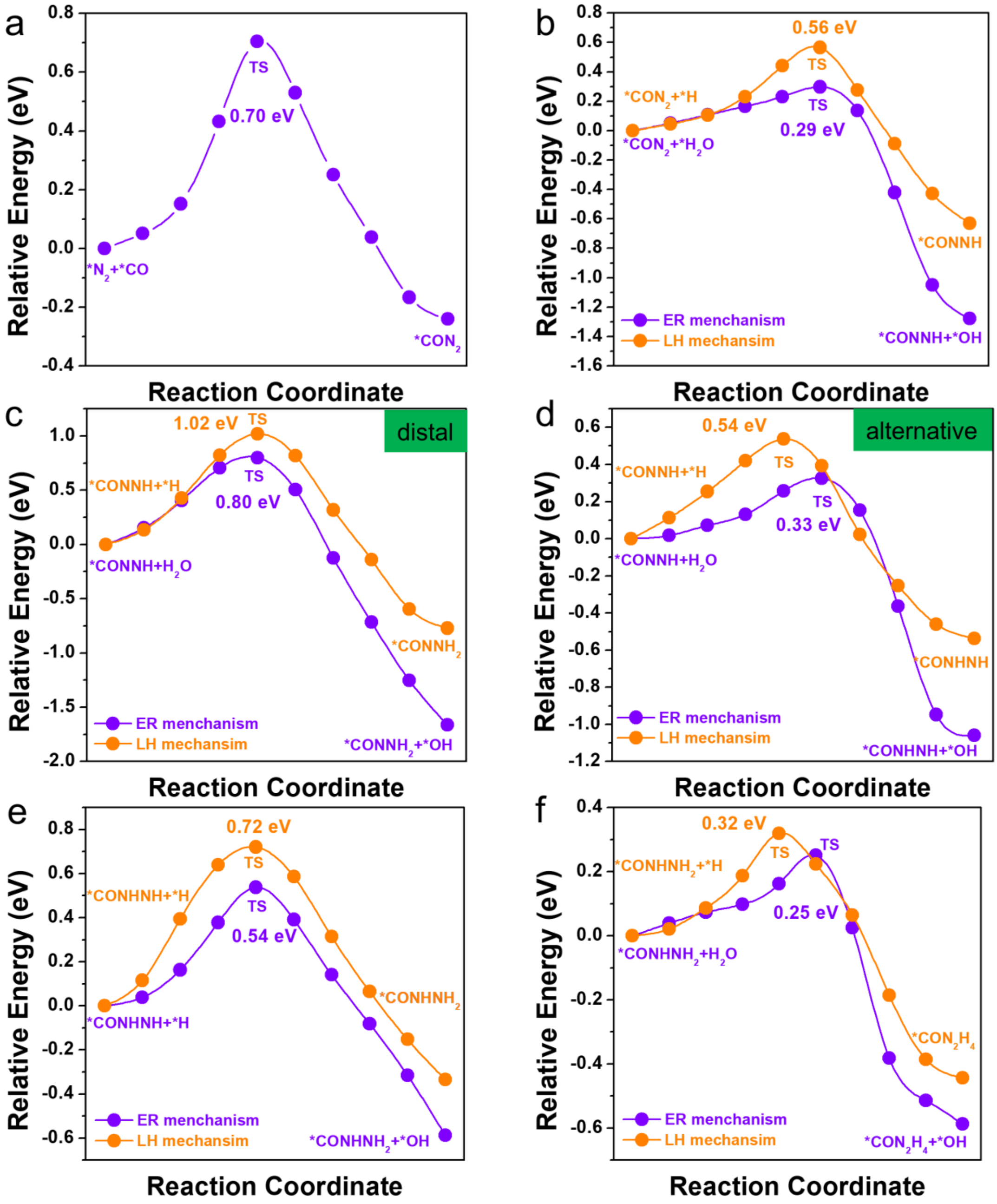

Supplementary Fig. 13 Kinetics of N_2 reduction to urea on Cr_2B_2. Kinetic pathways of elementary steps of N_2 reduction to urea on 2D Cr_2B_2.

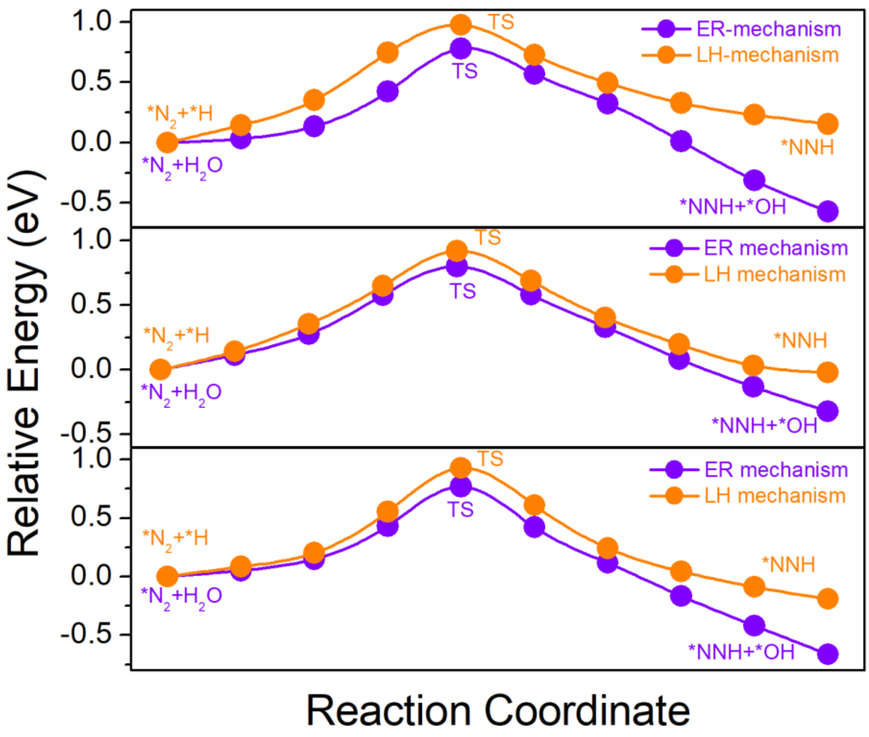

Supplementary Fig. 14 Kinetics of N_2 reduction to NH_3. Kinetic pathways for the reduction of N_2 to NNH on 2D Mo_2B_2, Ti_2B_2, and Cr_2B_2.

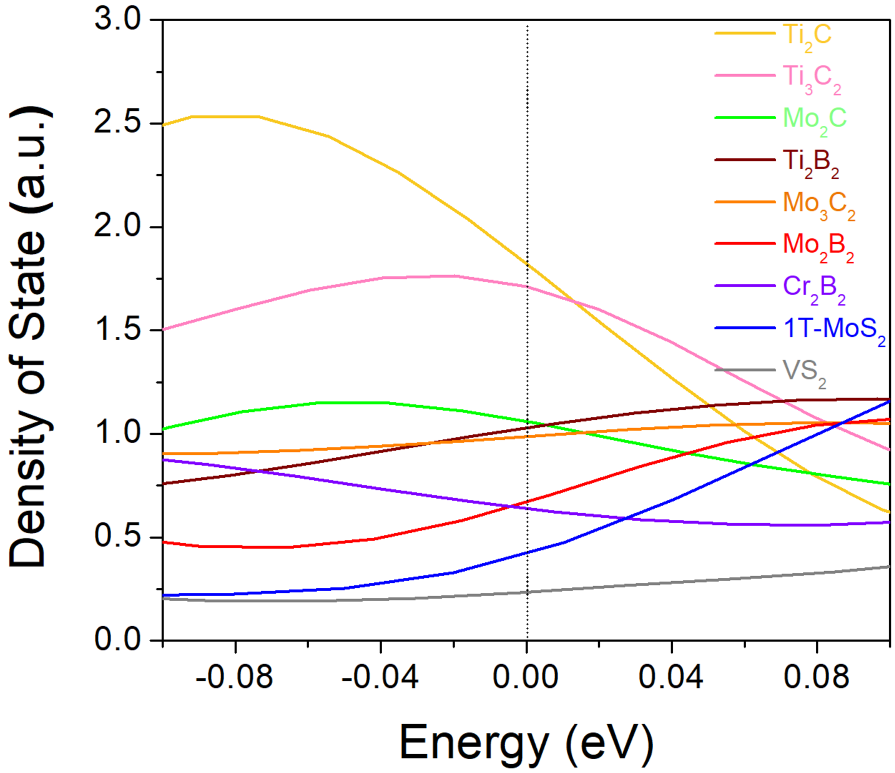

Supplementary Fig. 15 Specialty of MBenes for Urea Production. Comparison of electronic DOS (per atom) between MBenes and other 2D materials.

Supplementary Methods

Construction of Surface Pourbaix Diagrams The surface Pourbaix diagram is an effective tool to determine the stable surface states of the catalyst in electrochemical conditions as a function of pH and the electrode potential *U*. For an arbitrary M_2B_2 surface with adsorption site * in its pristine state, a generalized representation of the adsorption of oxygenated intermediates, denoted as O_mH_n*, can be written as:

$$*M_2B_2 - O_mH_n + (2m-n)(e^- + H^+) \rightleftarrows *M_2B_2 + mH_2O \quad (1)$$

where *m* and *n* are the number of adsorbed oxygen and hydrogen atoms, respectively. The associated free-energy change can be computed as：

$$\Delta G(U, pH) = G_{M_2B_2} + mG_{H_2O} - G_{*M_2B_2-O_mH_n} - (2m-n)(G_{e^-} + G_{H^+}) \quad (2)$$

According to the CHE model, the Gibbs free energy of proton/electron pairs can be represented as 1/2 H_2 on standard hydrogen electrode (SHE) scale:

$$G_{e^-} + G_{H^+} = \frac{1}{2}G_{H_2} - eU_{SHE} + k_BT(\ln[a_{H^+}]) \quad (3)$$

Then the free-energy change for adsorption of O_mH_n* intermediates can be written as:

$$\Delta G(U, pH) = G_{M_2B_2} + mG_{H_2O} - G_{*M_2B_2-O_mH_n} - (2m-n)(\frac{1}{2}G_{H_2} - U_{SHE} - 2.303k_BTpH) \quad (4)$$

According to above equations, we can derive a relation between potential and pH for a wide variety of oxygen-containing adsorbates on M_2B_2 in reference to standard conditions when ΔG (*U*, *pH*) = 0

Construction of Pourbaix Diagrams of 2D MBenes To map the whole Pourbaix diagrams of 2D MBenes, the formation energies of transition metals (Mo, Ti, Cr), bulk Boron (B), and standard O_2 and H_2 gases (298.15 K, 1.0 bar) were used as the references

to computed the Gibbs formation energies of the M_2B_2 derived compounds, while the formation energies of competing ionic and molecular species in solution were obtained from experimental database.[1] We expressed the relative chemical potentials μ^{ref} of considered species under any electrochemical condition using the Nernst equations. Therefore, the Gibbs free energy of each species containing elements *i = 1...n* considered in solution can be expressed with its formation energies at standard conditions G_i^0 using the reference states for the elements:

$$G_i^f = G_i^0 + 0.0591 \log c_i - n_O \mu_{H_2O} + pH(n_H - 2n_O) + \phi(2n_O - n_H + q_i) \quad (5)$$

Where c_i is the concentration of species *i*, a moderate concentration of 10^{-6} M were set for all ionic species. μ_{H2O} is the formation energy of water, while n_O and n_H are the containing oxygen and hydrogen atoms numbers in the species, ϕ is the electric potential, while q_i is the charge number of considered species, with this equation we can describes the Gibbs free energy of the solid phases as a function of pH and applied potential ϕ.

Microkinetic Simulations The equilibrium surface population of *OH or *CO at a given U was estimated by following equation:

$$\theta_*(U) = \frac{1}{Q(U)} \sum_i f_i \cdot e^{-\frac{\Delta G_i(U)}{k_B T}} \quad (6)$$

Where f_i is the coverage of *CO or *OH for a surface state *i*, ΔG_i (*U*) is the relative Gibbs free energy of state *i*, k_B is Boltzmann constant, *T* is room temperature (298.18 K), and *Q* is the partition function from all of the surface states considered.

Supplementary References

1. Pourbaix, M., Zhang, H. & Pourbaix, A. Presentation of an Atlas of chemical and electrochemical equilibria in the presence of a gaseous phase. *Mater. Sci. Forum* **251**, 143–148 (1997).
